# Eating behaviors in transmasculine and transfeminine adults assessed by the three factor eating questionnaire

**DOI:** 10.3389/fnut.2026.1671465

**Published:** 2026-02-18

**Authors:** John Michael Taormina, Matthew Bolt, Marc-Andre Cornier, Kerrie L. Moreau, Margaret E. Wierman, Micol S. Rothman, Kristina T. Legget, Jason R. Tregellas, Allison K. Hild, Daniel B. Hammond, Amanuail Gebregzabheir, Mary P. Mancuso, Mary D. Sammel, Sean J. Iwamoto

**Affiliations:** 1 Department of Family Medicine, University of Colorado Anschutz Medical Campus, Aurora, CO, United States; 2 Department of Biostatistics and Informatics, Colorado School of Public Health, University of Colorado Anschutz Medical Campus, Aurora, CO, United States; 3 Division of Endocrinology, Diabetes and Metabolic Diseases, Department of Medicine, Medical University of South Carolina, Charleston, SC, United States; 4 Division of Geriatrics, Department of Medicine, University of Colorado Anschutz Medical Campus, Aurora, CO, United States; 5 Division of Endocrinology, Metabolism and Diabetes, Department of Medicine, University of Colorado Anschutz Medical Campus, Aurora, CO, United States; 6 Department of Psychiatry, University of Colorado Anschutz Medical Campus, Aurora, CO, United States; 7 Division of General Internal Medicine, Department of Medicine, University of Colorado Anschutz Medical Campus, Aurora, CO, United States

**Keywords:** eating behavior, gender diverse, TFEQ, three-factor eating questionnaire, transgender

## Abstract

**Introduction:**

Transgender and gender diverse (TGD) people have unique influences on their eating and nutrition-related behaviors, which increase their risk for disordered eating. Understanding eating behaviors in this population may help healthcare providers screen for and treat eating pathologies. Few studies have evaluated their eating behavior traits using validated measurements.

**Methods:**

This secondary cross-sectional analysis evaluated eating behavior traits of transmasculine (TM) and transfeminine (TF) adults on testosterone-based and estradiol-based gender-affirming hormone therapy, respectively, for >1 year. The Three Factor Eating Questionnaire (TFEQ) was self-administered to assess the traits of cognitive restraint, disinhibition, and susceptibility to hunger. Linear regression models were used to estimate mean TFEQ scores to account for sample age variability, as well as estimate associations between BMI and BF% with TFEQ scores by gender group adjusted for age.

**Results:**

Of 16 participants in the TM group, actual mean scores (SD) were cognitive restraint 7.8 (4.3), disinhibition 6.7 (4.4), and susceptibility to hunger 5.9 (3.8). The actual mean scores (SD) of the 30 TF participants were cognitive restraint 10.2 (4.9), disinhibition 6.0 (3.9), and susceptibility to hunger 4.0 (3.6). The groups had similar actual TFEQ scores, though after adjustment for age, TF had a higher predicted mean cognitive restraint score while TM had a higher predicted mean susceptibility to hunger score. Eating behavior traits were generally positively associated with BMI and BF%. For TM, associations were positive between susceptibility to hunger for BMI and BF% and between disinhibition and BMI; meanwhile, for TF, associations were positive between cognitive restraint and BMI and BF%.

**Discussion:**

TM and TF had similar eating behavior traits assessed by the TFEQ that resemble TFEQ profiles previously reported for cisgender women but higher than previously reported for cisgender men. Associations of eating behavior traits with anthropometric measures of adiposity were generally positive, suggesting that TGD people with overweight or obesity may experience greater cognitive restraint, disinhibition, and susceptibility to hunger than TGD people with normal weight. Future larger studies should assess the relationships of these behaviors with other influences, such as disordered eating and gender minority stress, to further understand eating behaviors in TGD populations.

## 1 Introduction

Transgender and gender diverse (TGD) people are individuals with gender identities that are not aligned with their sex designated at birth (e.g., transgender men and women, nonbinary, genderqueer, gender nonconforming). TGD populations face significant mental and physical health disparities compared to their cisgender peers (1). These disparities are understood through the gender minority stress model, which proposes that health challenges for TGD individuals are the result of additional distal and proximal stressors imposed upon them as a result of their gender identity or expression (2). These additional challenges have prompted interest among healthcare providers and researchers in understanding and addressing the unique health barriers facing the TGD community.

An important but understudied challenge among TGD populations is nutrition and eating concerns (3–6). As a result of gender minority stress, TGD people have unique influences that affect their eating and nutrition-related behaviors. These include gender dysphoria and body dissatisfaction, societal pressures to conform to cisgender norms, and barriers to gender-affirming healthcare (7). Weight and shape control behaviors can lead to body changes that may align appearance more closely with gender identity, leading some TGD people to turn to disordered eating to reduce gender dysphoria, the psychological distress caused by misalignment of gender and sex designated at birth, and for societal acceptance or safety (8–10). TGD people may turn to these behaviors to address dysphoria when they are unable to access gender-affirming care due to discrimination from healthcare providers or structural delays in accessing care (e.g., long waitlists, body mass index [BMI] barriers). Consequently, eating disorders are prevalent among TGD populations; lifetime prevalence among transgender men and women is estimated to be 10.5% and 8.1%, respectively (11). Genderqueer and/or nonconforming individuals may be at even greater risk of disordered eating compared to transgender men and women (12). While this is understudied, these findings may represent minority stress faced by non-conforming individuals due to societal binary gender norms. Understanding the complexities of eating behaviors in TGD people may help healthcare providers more effectively screen for and manage eating and nutrition-related disorders in the TGD population.

Despite increasing recognition of the need to address eating and nutrition concerns for TGD people (3–6), there remains a lack of validated tools to evaluate their eating behaviors. The Three Factor Eating Questionnaire (TFEQ) (13) is a widely used tool to measure eating behavior traits. The original 51-item survey assesses (1) cognitive restraint, or conscious control of food intake to control weight; (2) disinhibition, or uninhibited food intake in response to non-hunger stimuli; and (3) susceptibility to hunger, or food intake in response to feelings or perceptions of hunger. Revised 21-item TFEQ (14, 15) and 18-item TFEQ (16) are available and differ from the original 51-item questionnaire in the factors assessed (i.e., cognitive restraint, uncontrolled eating, emotional eating) and the scoring (i.e., 4-point Likert scale scored rather than dichotomized scoring). Studies evaluating the TFEQ in cisgender cohorts have demonstrated sex differences (i.e., cisgender women have higher TFEQ scores than cisgender men) and differences based on body size (i.e., generally higher TFEQ scores for higher BMI) (14, 17, 18). A recent study of 84 TGD participants used the revised 18-item TFEQ, a Gender Variance Scale, and separate scales for self-perceived masculinity and femininity to evaluate eating differences based on gender expression and variance (19). This study found that masculinity and BMI were positively associated with cognitive restraint and femininity and BMI were positively associated with emotional and uncontrolled eating. Thirty-six participants used gender-affirming hormone therapy (GAHT) of variable duration (1 to 120 months), though eating behaviors did not differ by GAHT status or GAHT duration. To date, this is the only study evaluating eating behavior traits in a TGD cohort using any version of the TFEQ.

The present study assessed eating behavior traits using the original 51-item TFEQ in transmasculine adults (i.e., transgender men and nonbinary individuals on testosterone-based GAHT) and transfeminine adults (i.e., transgender women and nonbinary individuals on estradiol-based GAHT) after being on GAHT for greater than 1 year. The analyses contribute novel findings on this topic. First, the study evaluated eating behaviors by gender identity groups using either estradiol-based GAHT or testosterone-based GAHT for greater than 1 year rather than by gender variance or self-perceived expression. Furthermore, by using the original 51-item TFEQ, the analyses assessed in a TGD cohort the susceptibility to hunger and disinhibition eating factors, the latter being implicated in development of eating disorders and associated with body dissatisfaction (20). Finally, in addition to assessing differences in eating behavior associations with BMI, this study also assessed differences in TFEQ by total body fat percentage (BF%) as measured by dual-energy x-ray absorptiometry (DEXA), a direct measure of adiposity. While the results of this exploratory analysis should be interpreted as such, this study provides greater insight into the eating behaviors of TGD people to expand future research in this field.

## 2 Materials and methods

### 2.1 Study design

This study was a secondary analysis of cross-sectional data to (1) characterize the eating behavior traits of TGD adults taking GAHT for greater than 1 year using the TFEQ in the fasted state, and (2) compare these eating behavior traits in transmasculine adults on testosterone-based GAHT to those in transfeminine adults on estradiol-based GAHT. Retrospective data were collected from study protocols approved by the Colorado Multiple Institutional Review Board (COMIRB): two protocols including transfeminine cohorts (COMIRB 18-2258 and 20-2141) and one protocol including a transmasculine cohort (COMIRB 19-2323). All participants consented to participate in their respective studies.

The secondary analysis protocol to explore eating behavior traits among TGD adults was also reviewed and approved (COMIRB 25-1216). A waiver of consent was obtained during the approval process of the secondary analysis protocol. The secondary analysis was not a case–control study; therefore, analyses were conducted on all available data from the original studies and there was no matching between transmasculine and transfeminine adults for baseline characteristics. Data were collected and managed in the secure, web-based application designed to support data capture for research studies, REDCap (Research Electronic Data Capture), hosted at the University of Colorado Anschutz Medical Campus (21, 22).

### 2.2 Inclusion criteria

TGD participants were included if aged greater than or equal to 18 years and they were on estradiol- or testosterone-based GAHT (transfeminine adults: oral, transdermal, or parenteral estradiol with/without spironolactone and/or progesterone; transmasculine adults: transdermal or parenteral testosterone) for at least 1 year prior to the primary study and excluded if they had previously undergone gonadectomy, prior or active hormone-sensitive neoplasms, acute liver or gallbladder disease, venous thromboembolism, active/overt hyperthyroidism, current tobacco smoking (or quit smoking less than 1 year prior to the study), or illicit drug use.

### 2.3 Study visits

For each primary study, participants arrived at the research clinic in the morning after an overnight fast. The studies did not include a run-in diet due to budgetary constraints. All participants had the following data collected and assessed in the fasted state at the time of the primary studies: demographics (age, race, ethnicity, sex designated at birth, gender identity, and duration of GAHT), body measurements (weight in kilograms, height in centimeters), BMI (calculated by dividing measured weight by measured height squared), TFEQ scores, and total BF% measured by dual-energy x-ray absorptiometry (DXA, Horizon® W [S/N 300734M]). Serum estradiol was measured via chemiluminescent immunoassay (Beckman Coulter, sensitivity 10.0 pg./mL) and serum total testosterone was measured via 1-step competitive assay (Beckman Coulter, sensitivity 17.0 ng/dL).

The 51-item TFEQ was self-administered with 21 items to assess cognitive restraint, 16 items to assess disinhibition, and 14 items to assess susceptibility to hunger. The questionnaire included 36 yes/no questions and 14 items that were answered on a vertical rating scale of 1 to 4, all of which were dichotomized to 0 or 1. Each of the three factors was scored in aggregate separately, with higher scores corresponding to greater eating behavior traits. Participants answered the TFEQ questionnaire directly in REDCap as an individual instrument created by the research team and it was automatically scored by the REDCap program.

### 2.4 Statistical methods

Sample sizes for each group were determined by the available participants from the previous studies mentioned above. Continuous sample demographics were summarized with means and standard deviations and categorical sample demographics with counts and proportions, then stratified by TGD group. To estimate mean TFEQ scores accounting for age variability across TGD groups, linear regression models were fit with TFEQ scores as outcomes and with age and TGD group as independent variables. The resulting relevant covariates were then added together, assuming the mean sample age, to obtain group-level estimates. Linear regression models were also fit with the BMI or BF% as additional independent variables to estimate the associations between TFEQ scores and BMI or BF% by TGD group while adjusting for age. Regression model assumptions were assessed with standard diagnostic plots examining residuals, and potential leverage points. Model contrasts were utilized to estimate confidence intervals for these estimates. All regression modeling used transmasculine adults as the reference category. No adjustments were made for multiple comparisons, as *p*-values are not reported in this manuscript. A complete case approach was used to handle missing data. Missing data was due to data collection investigator error. Statistical analyses were conducted in R version 4.4.0 with associated packages (23–31). R code for the analysis is available upon request.

## 3 Results

### 3.1 Description of sample

The mean (standard deviation) age of the sample was 37.1 (14.5) years old; transmasculine adults were on average 30.1 (8.2) years old, and transfeminine adults 40.8 (15.9) years old. Most participants were White (89.1%) and Non-hispanic/Latino (88.9%). All transfeminine adults had a male sex designated at birth and all transmasculine adults had a female sex designated at birth. Mean BMIs were 25.3 (5.7) kg/m^2^ for transmasculine adults and 28.8 (5.7) kg/m^2^ for transfeminine adults. Mean BF% was 30.1 (7.0)% for transmasculine adults and 35.5 (7.9)% for transfeminine adults.

Among transfeminine adults (*n* = 30), two (7%) identified as nonbinary and the remainder identified as transgender women. Routes of current GAHT administration varied: oral estradiol (*n* = 13, 43%), transdermal patch (*n* = 7, 23%), estradiol valerate intramuscular injection (*n* = 6, 20%), sublingual estradiol (*n* = 2, 7%), estradiol valerate subcutaneous injection (*n* = 1, 3%), and estradiol cypionate intramuscular injection (*n* = 1, 3%). Most (*n* = 28, 93%) were taking spironolactone at the time of the study visit, while concurrent progesterone (*n* = 9, 30%) or finasteride (*n* = 2, 7%) were less common. The mean GAHT duration was 4.5 (4.2) years, serum estradiol was 189.6 (116.9) pg./mL, and serum total testosterone was 93.3 (111.0) ng/dL. Nineteen (63%) transfeminine participants were taking medications associated with weight gain, including anti-depressants (*n* = 14, 47%), progesterone (*n* = 9, 30%), and anti-histamines (*n* = 7, 23%). Additionally, 9 (30%) transfeminine participants were taking medications associated with weight loss, including stimulants (*n* = 4, 13%), bupropion (*n* = 3, 10%), and levothyroxine (*n* = 2, 7%).

Among transmasculine adults (*n* = 16), one identified as nonbinary and the rest identified as transgender men. Most (*n* = 9, 56%) used testosterone cypionate intramuscular injections at the time of the study visit, while others used testosterone cypionate subcutaneous injections (*n* = 4, 25%) or testosterone gel (*n* = 3, 19%). The mean GAHT duration was 4.7 (3.6) years, serum estradiol was 45.4 (23.9) pg./mL, and serum total testosterone was 502.9 (339.0) ng/dL. Seven (44%) were taking medications associated with weight gain, most commonly antidepressants (*n* = 3, 19%) and anti-histamines (*n* = 2, 13%), and only one participant (6%) was taking a medication associated with weight loss (bupropion).

Overall, the mean TFEQ cognitive restraint score was 9.3 (4.8) (out of 21), the mean TFEQ disinhibition score was 6.2 (4.1) (out of 16), and the mean TFEQ susceptibility to hunger score was 4.7 (3.7) (out of 14). For a full description of the sample, see Table 1. For visualization of BMI and BF% by group, see Supplementary Figure S1.

**Table 1:** Demographics and clinical characteristics.

Characteristic	Overall *N* = 46^1^	Transmasculine adults *N* = 16^1^	Transfeminine adults *N* = 30^1^
Age			
Mean (SD)	37.1 (14.5)	30.1 (8.2)	40.8 (15.9)
Race			
Non-white	5.0 (10.9)	3.0 (18.8)	2.0 (6.7)
White	41.0 (89.1)	13.0 (81.3)	28.0 (93.3)
Ethnicity			
Hispanic/Latino	5.0 (11.1)	2.0 (13.3)	3.0 (10.0)
Non-hispanic/Latino	40.0 (88.9)	13.0 (86.7)	27.0 (90.0)
Missing	1	1	0
Height (cm)			
Mean (SD)	171.9 (7.1)	165.6 (5.5)	175.3 (5.4)
Weight (kg)			
Mean (SD)	82.1 (20.5)	69.9 (18.0)	88.6 (18.9)
BMI			
Mean (SD)	27.6 (5.8)	25.3 (5.7)	28.8 (5.7)
BF%			
Mean (SD)	33.6 (7.9)	30.1 (7.0)	35.5 (7.9)
Missing	1	0	1
TFEQ cognitive restraint			
Mean (SD)	9.3 (4.8)	7.8 (4.3)	10.2 (4.9)
Median (Q1, Q3)	10.0 (5.0, 13.0)	7.0 (4.5, 10.5)	10.5 (7.0, 15.0)
TFEQ disinhibition			
Mean (SD)	6.2 (4.1)	6.7 (4.4)	6.0 (3.9)
Median (Q1, Q3)	5.0 (3.0, 10.0)	6.5 (2.5, 10.0)	5.0 (3.0, 7.0)
TFEQ susceptibility to hunger			
Mean (SD)	4.7 (3.7)	5.9 (3.8)	4.0 (3.6)
Median (Q1, Q3)	3.5 (2.0, 6.0)	5.0 (3.0, 8.5)	3.0 (2.0, 5.0)

1 *n* (%).

BMI, body mass index, BF%, total body fat percentage, IQR, interquartile range, SD, standard deviation, TFEQ, three factor eating questionnaire.

### 3.2 Mean TFEQ scores by gender accounting for age

In a model accounting for age, where calculations assumed a mean sample age of 37.1 years old, the mean TFEQ cognitive restraint score for transmasculine adults was 8.55 and transfeminine adults was 9.75. Mean TFEQ disinhibition scores accounting for age were 6.73 and 5.98 for transmasculine and transfeminine adults, respectively, while TFEQ susceptibility to hunger scores were 5.70 and 4.13, respectively. Confidence intervals for these estimates are shown in Table 2.

**Table 2:** Mean estimates of TFEQ scores by TGD group adjusting for age.

Gender	Cognitive restraint (score out of 21)		Disinhibition (score out of 16)		Susceptibility to hunger (score out of 14)	
	Estimate	95% CI	Estimate	95% CI	Estimate	95% CI
Transmasculine adults	8.55	[6.26, 10.84]	6.73	[4.61, 8.85]	5.70	[3.82, 7.58]
Transfeminine adults	9.75	[8.1, 11.4]	5.98	[4.47, 7.49]	4.13	[2.78, 5.48]

CI, confidence interval, TFEQ, three factor eating questionnaire.

### 3.3 Associations between TFEQ scores and BMI by TGD group

**Figure 1:**
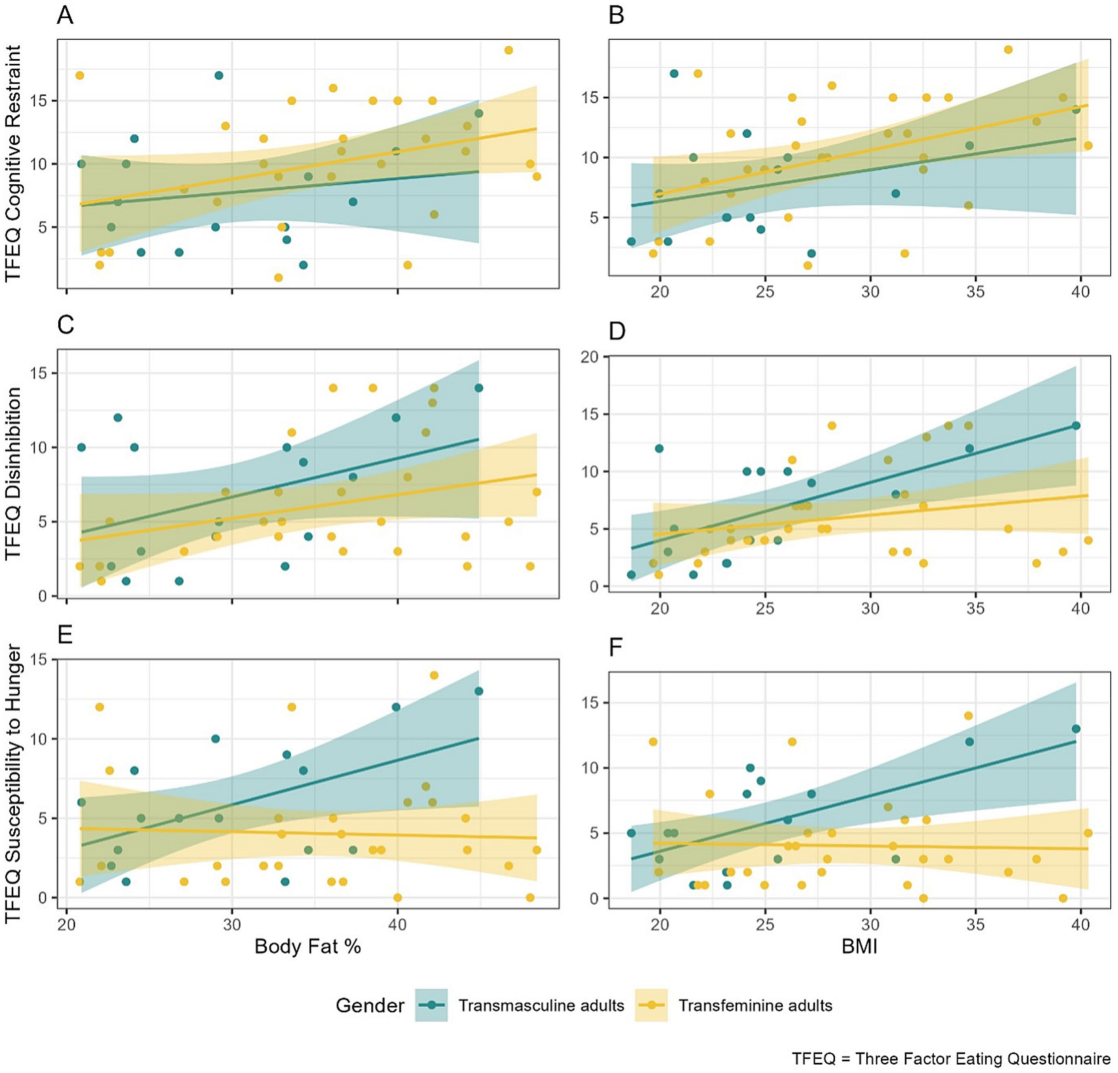
Associations between TFEQ scores and BF% and BMI. This figure shows associations of individual TFEQ factor scores with total BF% and with BMI. The first column shows associations between total BF% and **(A)** TFEQ cognitive restraint, **(C)** TFEQ disinhibition, and **(E)** TFEQ susceptibility to hunger. Similarly, the second column shows associations between BMI and **(B)** TFEQ cognitive restraint, **(D)** TFEQ disinhibition, and **(F)** TFEQ susceptibility to hunger. The *x*-axis displays total BF% or BMI (kg/m^2^), and the *y*-axis displays TFEQ scores (maximum 21 for TFEQ cognitive restraint, 16 for TFEQ disinhibition, and 14 for TFEQ susceptibility to hunger). Associations are shown separately by participant group: transmasculine adults (blue), and transfeminine adults (yellow). BF%, body fat percentage, BMI, body mass index.

Conditional relationships between TFEQ scores and BMI by TGD groups, adjusted for age, are shown in Table 3. For transmasculine adults, all outcomes were positively associated with BMI, though this trend was more evident for TFEQ disinhibition (0.51, 95% CI: [0.18, 0.84]) and TFEQ susceptibility to hunger (0.42, 95% CI [0.11, 0.73]). For transfeminine adults, cognitive restraint had the strongest association with BMI (0.36, 95% CI [0.09, 0.63]) and associations with disinhibition and susceptibility to hunger were weaker or non-existent. Visualizations of these relationships can be seen in Figure 1, and original model coefficients can be found in Supplementary Table S1.

**Table 3:** Regression coefficients (slopes) between BMI and outcomes by TGD group.

Gender	Cognitive restraint		Disinhibition		Susceptibility to hunger	
	Estimate	95% CI	Estimate	95% CI	Estimate	95% CI
Transmasculine adults	0.27	[−0.1, 0.64]	0.51	[0.18, 0.84]	0.42	[0.11, 0.73]
Transfeminine adults	0.36	[0.09, 0.63]	0.16	[−0.08, 0.4]	−0.02	[−0.24, 0.2]

CI, confidence interval, TFEQ, three factor eating questionnaire.

### 3.4 Associations between TFEQ scores and total BF% by TGD group

Conditional associations between TFEQ scores and BF% adjusted for age are shown in Table 4. For transmasculine adults, all TFEQ scores were at least weakly positively associated with BF%, though the only considerable association was with susceptibility to hunger (0.28, 95% CI [0.03, 0.53]). For transfeminine adults, cognitive restraint had a strong association with BF% (0.22, 95% CI [0.02, 0.42]), disinhibition was weakly associated with BF%, and there was no association between susceptibility to hunger and BF%. Figure 1 visualizes these relationships, and original model coefficients can be found in Supplementary Table S1.

**Table 4:** Regression coefficients (slopes) between body fat percentage by TGD group.

Gender	Cognitive restraint		Disinhibition		Susceptibility to hunger	
	Estimate	95% CI	Estimate	95% CI	Estimate	95% CI
Transmasculine adults	0.11	[−0.2, 0.42]	0.26	[−0.03, 0.55]	0.28	[0.03, 0.53]
Transfeminine adults	0.22	[0.02, 0.42]	0.16	[−0.04, 0.36]	−0.02	[−0.2, 0.16]

CI, confidence interval, TFEQ, three factor eating questionnaire.

## 4 Discussion

This study evaluated eating behavior traits and their associations with anthropometric measurements in transmasculine adults on testosterone-based GAHT and transfeminine adults on estradiol-based GAHT. Novel contributions of this study include disinhibition and susceptibility to hunger scores in a TGD cohort, associations of these factors and cognitive restraint with BMI and BF%, and evaluation of these data by gender identity group supported by GAHT. While exploratory, the results suggest that gender identity is a characteristic that should be considered when assessing TFEQ results, and associations with BMI and BF% are generally positive regardless of gender identity group but strength of associations may vary by sample.

### 4.1 TFEQ scores

The TGD cohort in this study had higher TFEQ cognitive restraint scores (mean score 9.3) with similar TFEQ disinhibition (mean score 6.2) and hunger scores (mean score 4.7) compared to other populations previously studied (32–35). In these earlier studies in presumed cisgender participants, mean cognitive restraint was in the range of 6–8, mean disinhibition 4.5–6.5, and susceptibility to hunger 4–6. In their original work, Stunkard & Messick had proposed cut-offs for “high scores” for TFEQ cognitive restraint (>10), disinhibition (>8), and susceptibility to hunger (>7) (13). However, the aforementioned studies have suggested that using the median split in the sample under investigation may be more appropriate than the original cut-offs and are typically lower (32–35). While the present study’s results are exploratory, applying the median split would lower cut-off scores for transmasculine adults for cognitive restraint (7.0), but not for transfeminine adults (10.0), and would lower cut-off scores for both transmasculine and transfeminine adults for disinhibition (6.5 and 5.0, respectively) and for susceptibility to hunger (5.0 and 3.0, respectively).

Assessing results by gender, transfeminine and transmasculine adults had overall similar profiles to cisgender women and higher scores compared to cisgender men in prior studies, particularly in disinhibition and cognitive restraint scores. These findings may reflect the increased eating disorder risk in transgender populations and cisgender women compared to cisgender men (36). In comparison to the only other study using any version of the TFEQ in a TGD cohort, Meneguzzo, et al., found associations between self-perceived masculinity and cognitive restraint and self-perceived femininity and emotional and uncontrolled eating, which are eating factors in the revised 18-item TFEQ that were derived from the original disinhibition and susceptibility to hunger factors. While the present study did not assess self-perceived masculinity/femininity, the results appear to be discordant with the prior study given the higher cognitive restraint scores in transfeminine adults and higher susceptibility to hunger scores in transmasculine adults. This discrepancy may be related to differences in the samples evaluated: the prior study included 10 nonbinary individuals and only 43% of the sample was on GAHT compared to 3 nonbinary participants and 100% on GAHT in the present study. Thus, the TGD groups evaluated in the present study may be more homogenous than the prior study and do not adequately reflect the eating behaviors of TGD adults that are not using GAHT and nonbinary adults. Future studies should include greater numbers of nonbinary adults to better characterize this TGD groups’ unique eating behavior traits.

The differences between TFEQ scores in TGD groups in the present study may be explained by GAHT use and type. While both TGD groups had high-normal disinhibition scores, transfeminine adults on estradiol-based GAHT had relatively higher cognitive restraint compared to transmasculine adults on testosterone-based GAHT. The higher relative cognitive restraint in transfeminine adults may reflect greater risk of eating disorder severity and eating control dysregulation, which have been associated with the combination of high disinhibition and very high cognitive restraint scores (20). This explanation is supported by recent findings that GAHT use is associated with increased eating disorder risk in transfeminine adults and reduced risk in transmasculine adults (37). GAHT use may also explain higher susceptibility to hunger scores in transmasculine adults compared to transfeminine adults; testosterone and estrogen increase and decrease appetite, respectively, through central mechanisms (38). Given the known effects of estrogen and testosterone on food intake (39–41), the results of these analyses must be interpreted only in the context of the sample studied and should not be extrapolated to TGD cohorts without GAHT use. The current study was not designed to evaluate the association of eating behavior traits with disordered eating symptomatology or hormone status, and further studies are warranted to evaluate these associations.

### 4.2 Associations of TFEQ with BMI and BF%

Though statistical significance was not assessed due to the small sample size of this study, the relationships between most TFEQ factors and BMI and BF% were positive. The only exception was the flat association between TFEQ susceptibility to hunger and BMI and BF% for transfeminine adults. Generally, these results are in line with prior studies of presumed cisgender cohorts that have established a positive association for BMI with disinhibition and with susceptibility to hunger; however, prior studies evaluating the relationship with cognitive restraint have demonstrated mixed results, with some studies suggesting a potential quadratic relationship (33, 42–46). The results of the present study are also in line with those of Meneguzzo et al. (19) which demonstrated that TGD participants had significant positive associations between BMI and TFEQ cognitive restraint, uncontrolled eating, and emotional eating. These results suggest that cognitive restraint, dysregulated eating and susceptibility to hunger may be more prominent in TGD adults with higher BMI.

This is the first study to analyze the association of eating behavior traits with BF% measured by DEXA in a TGD cohort. Unlike BMI, DEXA is a direct measure of adiposity. In a TGD cohort on GAHT, assessing the association of eating behavior traits with BF% may be more informative than BMI as estradiol- and testosterone-based GAHT are associated with weight gain with differential effects on body composition; estradiol-based GAHT is associated with increased adiposity and decreased lean mass and the opposite body composition changes are associated with testosterone-based GAHT (47–50). The present study suggested that transfeminine adults had higher BF% than transmasculine adults, consistent with body composition changes associated with respective GAHT. While most associations trended positively, the most evident positive findings were with cognitive restraint and BMI/BF% for transfeminine adults and with susceptibility to hunger and BMI/BF% and with disinhibition and BMI only for transmasculine adults. The results of the present study are exploratory, and assessment of significant associations are limited by the small sample size. However, the associations for BF% largely trended similarly to the associations for BMI.

In comparison, few other studies have evaluated this association in presumed cisgender cohorts and none to date have used DEXA. One study of 116 young cisgender women in New Zealand with body composition measured using air displacement plethysmography (BodPod®) demonstrated that BF% was positively associated with disinhibition but not associated with cognitive restraint nor susceptibility to hunger (43). Another recent study of 175 young American cisgender women and men using bioelectrical impedance (InBody®) and the 18-item revised TFEQ found that BF% was positively associated with cognitive restraint and emotional eating in both sexes (51). Additionally, a study of 596 French Canadian cisgender women and men with body composition measured by hydrostatic weighing found that cisgender women and men had significant positive correlations for disinhibition and susceptibility to hunger but not cognitive restraint (52). When cognitive restraint was further categorized into rigid and flexible restraint subscales, cisgender women were found to have a significant negative association with flexible restraint and a significant positive association with rigid restraint (53). It is difficult to compare these studies to each other and to the present study as each used a different method of determining BF%, and none of the methods except DEXA are direct measures of adiposity. However, these conflicting results suggest that the association between eating behavior traits and BF% may be more complex than the association for BMI, and future studies may consider using TFEQ subscales such as rigid and flexible restraint to better characterize the associations of eating behavior traits and body fat in TGD cohorts.

### 4.3 Limitations

This exploratory study has multiple limitations. Foremost is the small sample size, particularly of transmasculine adults. The small sample size limits the generalizability of the results and limited the ability to assess significant associations with eating behavior traits. This is a secondary analysis of primary data collected from available TGD samples and thus the samples were not matched for age, BMI or BF%. Importantly, the mean age of the transfeminine adults was 10 years higher than transmasculine adults, which could have influenced the results. Additionally, gender diverse identities were not separated out from transgender men and transgender women in the analysis given the small number of these individuals: 2 transfeminine nonbinary adults on estradiol-based GAHT and 1 transmasculine nonbinary adult on testosterone-based GAHT. Thus, differences between nonbinary and transgender individuals on GAHT cannot be assessed, and this study cannot speak to the nonbinary experience. Future research should include adequate representation of nonbinary individuals on GAHT to better understand eating behaviors in nonbinary individuals. Furthermore, the participants completed the original 51-item TFEQ, which limited the assessment to only the three main factors: cognitive restraint, disinhibition, and susceptibility to hunger. Subscales of cognitive restraint (e.g., rigid and flexible control of eating) and of disinhibition and susceptibility to hunger (e.g., uncontrolled eating and emotional eating) can further characterize eating behavior traits with revised and modified versions of the TFEQ (17). The studies used in this analysis also did not assess for other measures such as disordered eating or gender minority stress, which may have allowed for more nuanced interpretation of the results. Finally, the cross-sectional design of this study limits assessment of these scores on these and other eating and nutrition-related outcomes and the impact of GAHT on these outcomes over time in TGD adults compared to their pre-GAHT baseline.

## 5 Conclusion

This is the first study to evaluate cognitive restraint, disinhibition, and susceptibility to hunger using the original 51-item TFEQ in a cohort of transmasculine adults on testosterone-based GAHT and transfeminine adults on estradiol-based GAHT, while also assessing the association of eating behavior traits with both BMI and BF%. Transmasculine and transfeminine adults had similar TFEQ profiles to what has been recorded in other published cohorts of cisgender women and generally higher scores compared to previously recorded scores of cisgender men. Associations of TFEQ with BMI and BF% were generally positive or trended positively, particularly for cognitive restraint and disinhibition, suggesting that TGD people with overweight or obesity may experience these eating behavior traits to a greater degree than those with lower body weights.

While this study is exploratory, the results help further the understanding of eating behaviors in transmasculine and transfeminine adults and may have implications for clinical use by healthcare practitioners caring for TGD patients. The results suggest that assessing for cognitive restraint and disinhibition in nutritional assessments of TGD individuals, particularly those with higher BMI and BF%, may facilitate detection of disordered eating behaviors or those at risk. Future research should assess the relationships of these behaviors with other metrics such as measures of disordered eating, gender dysphoria, and gender minority stress, to better characterize the mechanisms underlying eating behaviors and eating and nutrition-related pathology in TGD populations.

## Data Availability

The raw data supporting the conclusions of this article will be made available by the authors, without undue reservation.
