# Supplementary material for: Eating behaviors in transmasculine and transfeminine adults assessed by the three factor eating questionnaire
Source: Front Nutr. 2026 Feb 18;13:1671465. doi: 10.3389/fnut.2026.1671465 (PMC12956684; doi:10.3389/fnut.2026.1671465)
Supplement: Supplementary file 1 [file Table_1.docx]

**Supplemental Table 1: Regression model equations**

|  | **TFEQ Cognitive Restraint** | | **TFEQ Disinhibition** | | **TFEQ Susceptibility to Hunger** | |
| --- | --- | --- | --- | --- | --- | --- |
| **term** | **Est** | **95% CI** | **Est** | **95% CI** | **Est** | **95% CI** |
| (Intercept) | 4.35 | [0.61, 8.08] | 6.52 | [3.06, 9.97] | 6.62 | [3.56, 9.68] |
| Transfeminine | 1.20 | [-1.79, 4.19] | -0.75 | [-3.51, 2.01] | -1.57 | [-4.02, 0.87] |
| Age (years) | 0.11 | [0.01, 0.21] | 0.01 | [-0.09, 0.1] | -0.02 | [-0.11, 0.06] |


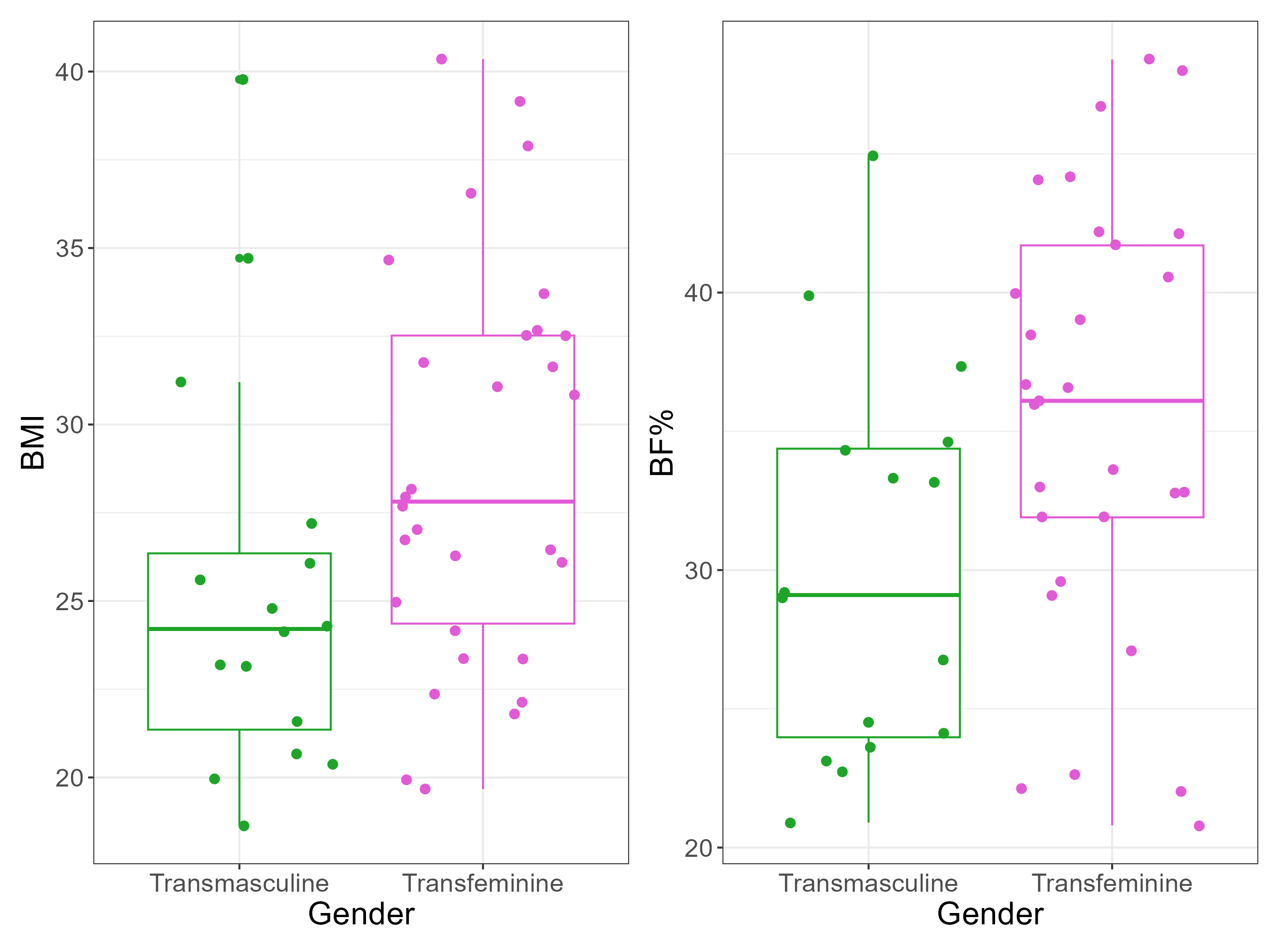


**Supplemental Figure 1: Distribution of BMI and BF% by Gender**
